# Short Term Traffic State Prediction via Hyperparameter Optimization Based Classifiers

**DOI:** 10.3390/s20030685

**Published:** 2020-01-27

**Authors:** Muhammad Zahid, Yangzhou Chen, Arshad Jamal, Muhammad Qasim Memon

**Affiliations:** 1 College of Metropolitan Transportation, Beijing University of Technology, Beijing 100124, China; zahid@emails.bjut.edu.cn; 2 College of Artificial Intelligence and Automation, Beijing University of Technology, Beijing 100124, China; 3 Department of Civil and Environmental Engineering, King Fahd University of Petroleum & Minerals, KFUPM Box 5055, Dhahran 31261, Saudi Arabia; arhad.jamal@kfupm.edu.sa; 4 Advanced Innovation Center for Future education, Faculty of Education, Beijing Normal University (BNU), Beijing 100875, China; memon_kasim@bnu.edu.cn

**Keywords:** traffic state prediction, spatio-temporal traffic modeling, simulation, machine learning, hyper parameter optimization, ITS

## Abstract

Short-term traffic state prediction has become an integral component of an advanced traveler information system (ATIS) in intelligent transportation systems (ITS). Accurate modeling and short-term traffic prediction are quite challenging due to its intricate characteristics, stochastic, and dynamic traffic processes. Existing works in this area follow different modeling approaches that are focused to fit speed, density, or the volume data. However, the accuracy of such modeling approaches has been frequently questioned, thereby traffic state prediction over the short-term from such methods inflicts an overfitting issue. We address this issue to accurately model short-term future traffic state prediction using state-of-the-art models via hyperparameter optimization. To do so, we focused on different machine learning classifiers such as local deep support vector machine (LD-SVM), decision jungles, multi-layers perceptron (MLP), and CN2 rule induction. Moreover, traffic states are evaluated using traffic attributes such as level of service (LOS) horizons and simple if–then rules at different time intervals. Our findings show that hyperparameter optimization via random sweep yielded superior results. The overall prediction performances obtained an average improvement by over 95%, such that the decision jungle and LD-SVM achieved an accuracy of 0.982 and 0.975, respectively. The experimental results show the robustness and superior performances of decision jungles (DJ) over other methods.

## 1. Introduction

Smart cities have emerged at the heart of “next stage urbanization” as they are equipped with fully digital infrastructure and communication technologies to facilitate efficient urban mobility. The fundamental enabler of a smart city is dependent on connected devices, though the real concern is how the collected data are distributed city-wide through sensor technologies via the Internet of Things (IoT). Heterogeneous vehicular networks in a connected infrastructure network are able to sense, compute, and communicate information through various access technologies: Universal Mobile Telecommunications System (UTMS), Fourth Generation (4G), and Dedicated Short-Range Communications (DSRC) [1,2]. In vehicular sensor networks (VSN) and Internet of vehicles (IOV), each vehicle act as receivers, senders, and routers simultaneously to transmit data over the network or to a central transportation agency as an integral part of intelligent transportation systems (ITS) [3,4]. Furthermore, each and every network node in VSN is assumed to store, carry, and precisely transfer the data with cooperative behavior. In recent years, following rapid diversification, navigation technologies and traffic information services enable a large amount of data to be collected from the different devices such as loop detectors, on-board equipment, speed sensors, remote microwave traffic sensors (RTMS), and road-side surveillance cameras etc., that have been proactively used for monitoring of traffic conditions in the ITS domain [5,6,7,8,9]. Sensor networks in the form of road side units (RSUs) offer numerous applications including broadcasting periodic informatory, warnings, and safety messages to road users. The data obtained from these different sources have provided myriad opportunities to estimate and predict travel time and future traffic states through a large number of data-driven computational and machine learning approaches. Accurate traffic state prediction (TSP) ensures efficient vehicle route planning, and pro-active real-time traffic management.

TSP is achieved in three distinct steps: (i) prediction of the desired traffic flow parameters (i.e., volume, speed, and occupancy); (ii) identification of traffic state; and (iii) realizing the traffic state output. TSP can be classified as either short-term prediction or long-term prediction. In the former prediction type, short-term changes in traffic status are predicted (e.g., during a 5, 10, 15, or 30 min prediction horizon), and long-term prediction is usually estimated in days and months [10]. Short-term predictions can either be used directly by traffic professionals to take appropriate actions or can be added as inputs for proactive solutions in congestion management. Short-term prediction reduces common problems such as traffic congestion, road accidents, and air pollution; meanwhile, it also offers road users and traffic management agencies with important information to assist in better decision-making [11]. Three factors affect the quality of prediction in real-time traffic information. These factors include: (i) variation in data collected from various sources like sensors and other sources; (ii) dynamic nature of traffic conditions; and (iii) randomness and stochastic nature of traffic appearing in the supply and demand. However, addressing these factors remained challenging and significant in the realm of quality prediction for real-time traffic information [12].

In TSP, prediction methodologies are broadly studied into two main categories: parametric and non-parametric techniques [8]. Parametric methods include auto aggressive integrated moving average method ARIMA [13], exponential smoothing (ES) [14], and seasonal auto aggressive integrated moving average method (SARIMA) [15,16]. In their study, Li et al. suggested that a multi-view learning approach estimates the missing values in traffic-related time series data [17]. Parametric methods focus on pre-determining the structure of the model based on theoretical or physical assumptions, later tuning a set of parameters that represent the traffic conditions (i.e., a trend in the actual world) [10,11]. These practices develop a mathematical function between historical and predicted states, for instance, model-based time series such as ARIMA, which is commonly used for traffic predictions in all parametric methods [18]. However, autoregressive models provide better accuracy for TSP models, while considering the traffic information about upstream and downstream locations is accounted for on freeways [9]. Parametric methods have good accuracy and high computational efficiency and are highly suited for linear or stationary time-series [19]. On the other hand, non-parametric approaches provide several advantages such as the ability to avoid model’s strong assumptions and learn from the implicit dynamic traffic characteristics through archived traffic data. These models have the benefit of being able to manage non-linear, dynamic tasks, and can also utilize spatial–temporal relationships, whereas non-parametric methods require a large amount of historical data and training processes. Non-parametric techniques include artificial neural network (ANN) [20,21,22]; support vector regression (SVR) [23,24]; K-nearest neighbor (KNN) [25,26,27,28,29]; and Bayesian models [30,31]. Since non-parametric techniques yield better prediction accuracy compared to ordinary parametric techniques like time series as they require significantly high computational effort. Their prediction accuracy is largely dependent on the quantity and quality of training data [32]. The above-mentioned methods have been successfully deployed in various transport related applications where predictions are required for excessive passenger flow at a metro station or in a crowd gathered for a special event [33,34].

A critical review of literature for TSP indicates that time series and conventional ANN models have been widely employed for short term TSP. Although these models were aimed to fit the speed, density, or volume data as they usually inherit an overfitting issue. Thereby, the ability of models that capture generalized trends for traffic prediction is compromised. Macroscopic traffic parameters such as traffic flow, traffic speed, and density are the state variables of interest used in TSP, and are subsequently evaluated using level of service (LOS). However, training and testing the accuracy for the majority of such modeling approaches is frequently questioned. To overcome this issue, we incorporated recent AI and machine learning state-of-art-approaches such as decision jungles and LD-SVM (via hyperparameter optimization) as these methods have been rarely been explored in the existing works. Data utilized in current study was extracted from traffic simulator ‘VISSIM’, which realistically simulates complex vehicle interaction in transportation systems. Furthermore, this study has major contributions in terms of spatiotemporal analysis of different LOS classes (i.e., A-F) under different data-collection time-intervals. In general, we emphasized short-term prediction, which is considered useful for improving the productivity of transportation systems, and also beneficial in reducing both the direct and indirect costs. Moreover, this study reviewed the different techniques and approaches that have been used for short-term TSP. A comprehensive comparative analysis was also conducted to evaluate the ability and efficiency of proposed methods in terms of prediction accuracy. The specific main contributions of this paper are:

- We extend the exploration of decision jungles and locally deep SVM (LD-SVM) for short term traffic state prediction using hyperparameter optimization (via random sweep).
- A comprehensive comparison was implemented to demonstrate the ability and the effectiveness of each machine learning model for TSP accuracy.
- Prediction performances were evaluated under different forecasting time-intervals at distinct time scales.
- Short-term traffic state was taken as a function of level of service (LOS) along a basic freeway segment. Study results demonstrated that decision jungles were more efficient and stable at different predicted horizons (time-intervals) than the LD-SVM, MLP, and CN2 rule induction.

The remainder of this paper is organized as follows. Section 2 presents a brief overview of the methods and techniques for TSP in the existing literature. Section 3 describes the preliminaries for different machine learning models used in this study. Section 4 presents study area, data description, and key parameter settings. Section 5 highlights results and discussion. Section 6 includes the comparison of different models. Finally, Section 7 summarizes the conclusions, presents key study limitations, and outlook for future studies.

## 2. Related Work

Since early 1980, non-linear traffic flow prediction has been the focus of several research studies as it is regarded as extremely useful for real-time proactive traffic control measures [15,16]. From its inception in the 1980s, artificial neural networks (ANNs) have been widely used for the analysis and prediction of time series data. They have the ability to perceive the non-linear connection between features of input and output variables that in turn can produce effective TSP solutions. For example, Zheng et al. combined Bayesian inference and neural networks to forecast future traffic flow [35]. Ziang and Adeli proposed a time-delay via recurrent wavelet neural network, where the periodicity demonstrated the significance of traffic flow forecasting [36]. Parametric methods can obtain better prediction outcomes when the data flow of the traffic varies temporally. These methods assume a variety of difficult conditions such as residual normalization and predefined system structure and rarely converged due to the stochastic or non-linear traffic flow characteristics.

To address the limitations of parametric models, different approaches including linear kernel, polynomial kernel, Gaussian kernel, and optimized multi kernel SVM (MK-SVM) have been proposed by recent research studies for traffic flow prediction [37,38,39,40]. MK-SVM predicted the results by mapping the linear parts of historical traffic flow data using the linear kernel, while map residual was performed using the non-linear kernel. Alternatively, generating if–then rules, also known as rule induction techniques that search the training data for proposition rules, can also be used. which CN2 is best-known example of this approach, that have been successfully utilized by previous for flow prediction [41,42]. Hashemi et al. developed different models for classification based on if–then rules in the short-term traffic state prediction for a highway segment [43]. In contrast, ANNs’ popular network structure is multi-layer perceptron (MLP), which has been widely used in many transport applications due to its simplicity and capacity to conduct non-linear pattern classification and function approximation. The MLP model generally works well in the capture of complex and non-linear relations, but it usually requires a large volume of data and complex training. Many researchers, therefore, consider it as the most commonly implemented network topology [44,45,46]. Recently, in the study by Chen et al., they adapted a novel approach using dynamic graph hybrid automata for the modeling and estimation of density on an urban freeway in the city of Beijing, China [47]. The authors validated the feasibility of their modeling approach on Beijing’s Third Ring Road. A recent study conducted by Zahid et al., proposed a new ensemble-based Fast forest quantile regression (FFQR) method to forecast short-term travel speed prediction [48]. It was concluded that proposed approach yielded robust speed prediction results, particularly at larger time-horizons.

Aside from the above-mentioned models, decision trees and forests have a rich history in machine learning and have shown significant progress in TSP, as reported in some of the recent literature [49,50]. Various studies have been conducted to address the shortcomings of traditional decision trees, for example, their sub-optimal efficiency and lack of robustness [51,52]. Similarly, in another research study, the researchers investigated the efficacy of the ensemble decision trees for the TSP [50]. It was concluded that trees generate efficient predictions traditionally. At the same time, researchers have concluded that learning with ideal decision trees could be problematic due to overfitting [53]. Henceforth, this approach has some limitations, such that the amount of data to be provided as the number of nodes in decision trees would increase exponentially with depth, affecting the accuracy [54]. Recently, a study proposed a novel online seasonal adjustment factors coupled with adaptive Kalman filter (OSAF-AKF) model for estimating the real-time seasonal heteroscedasticity in traffic flow series [55].

In contrast, machine learning techniques and their performances for classifying different problems have been encouraging such as decision jungles and LD-SVM, which are heavily dependent on a set of hyperparameters that, in turn, efficiently describes different aspects of algorithm behavior [54,56,57]. It is important to note that no suitable default configuration exists for all problem domains. Optimizing the hyperparameter for different models is important in achieving good performance in the realm of TSP [56]. There are two types of hyperparameter optimization: manual and automatic. Manual is time-consuming and depends on expert inputs, while an automatic approach removes expert input. Automatic approaches include the most common practice methods such as grid search and random search [58]. Several libraries have recently been introduced to optimize hyperparameters. Hyperopt Library is one of the libraries offering different hyper-optimization algorithms for machine learning algorithms [59]. Existing techniques for optimizing EC-based hyperparameters [60,61] such as differential evolution (DE) and particle swarm optimization (PSO) are useful since they are conceptually easy and can achieve highly competitive output in various fields [62,63,64,65]. However, these methods have a great deal of calculation and a low convergence rate in the iterative process. In contrast, hyperparameter optimization methods such as random grid, entire grid, and random sweep have achieved a great deal of attention in hyperparameter optimization. In a random grid, the matrix is computed for all combinations, and the values are extracted from the matrix by the number of defined iterations in relation to the entire grid incurred for all possible combinations. The difference between the random grid and the random sweep is that the latter technique selects random parameter values within the set, while the former only employs the exact values defined in the algorithm module. With this understanding, random sweep was chosen for the models conducted in this study for hyperparameter optimization with the intention of improving the accuracy of short-term TSP.

## 3. Preliminaries

Machine learning provides a number of supervised learning techniques for classification and prediction. The objective of a classification problem is to learn a model, which can predict the value of the target variable (class label) based on multiple input variables (predictors, attributes). This model is a function, which maps as an input attribute vector X to the output class label (i.e., Yϵ {C1, C2, C3, …, Cn}). The label training set is represented as follows:

(1) $$(X,Y)=\left\{(x_{0},\;x_{1},\;x_{2},\;x_{3},\;\ldots x_{n}),Y\right\}$$

where Y is the target label class (dependent variable) and vector X is composed of *x*_0_, *x*_1_, *x*_2_, *x*_3_, …, *x_n_.* The macroscopic flow, density, and speed obtained from traffic simulation are referred to as input parameters when fed/imported to machine learning models for short term traffic prediction. The model learns from these input variables for different time intervals (i.e., 5, 10, and 15 min). Either the next time interval level of service (LOS) is considered as a class label or target variable. The predicted label class for time (Time duration = 1), is given in the following form:

(*Density*_1_, *Speed*_1_, *Flow*_1_, *Time Duration*_1_, *LOS*_2_) (2)

The current study utilized four different machine learning methods for short term TSP. These methods included LD-SVM, decision jungles, CN2 rule induction, and MLP. The detailed methodology for each technique is presented below. 

### 3.1. Local Deep Support Vector Machine (LD-SVM)

**Figure 1:**
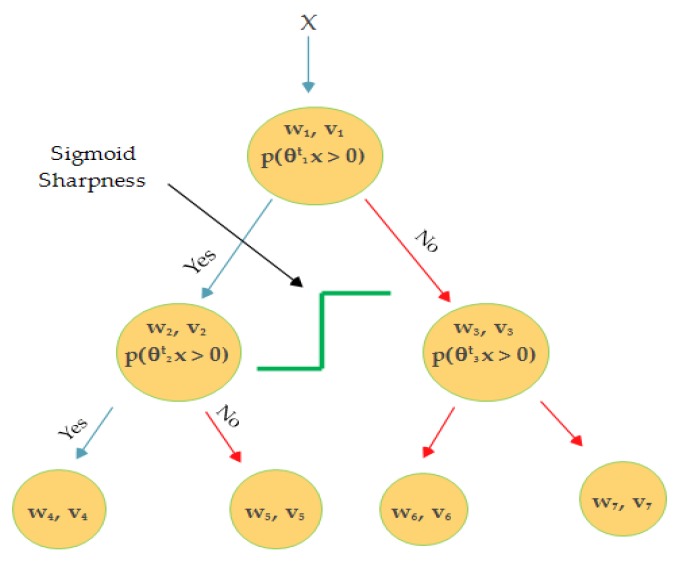
Schematic diagram of the as local deep support vector machine (LD-SVM).

SVM is based on statistical learning theory as suggested by Vapnik in 1995 for classification and regression [66]. Local deep kernel learning SVM (LD-SVM) is a scheme for effective non-linear SVM prediction while preserving classification precision above an acceptable limit. Using a local kernel function allows the model to learn arbitrary local embedding features including sparse, high-dimensional, and computationally deep features that bring non-linearity into the model. The model employs routines that are effective and primarily infused to optimize the space of local tree-structured embedding features in more than half a million training points for big training sets. LD-SVM model training is exponentially quicker than traditional SVM models training [57]. LD-SVM can be used for both linear and non-linear classification tasks. It is considered as a special type of linear classifier (e.g., logistic regression LG), however, LG is unable to perform sufficiently in complicated and linear tasks. In addition, LD-SVM model learning is significantly faster and computationally more efficient than traditional SVM model training. The formulation of a local deep kernel learns a non-linear kernel $K\;\left(x_{i},\;x_{j}\right)=K_{L\;}\left(x_{i},\;x_{j}\right)\;K_{G}\left(x_{i},\;x_{j}\right)$, where $K_{L}$ and $K_{G}$ are the local and global kernel. The product of local kernel $K_{L\;}=\;\phi_{L}^{t}\phi_{L}$ and global kernel $K_{G\;}=\;\phi_{G}^{t}\phi_{G}$ leads to the prediction function.

(3) $$y\left(x\right)=sign\left(\phi_{L}^{t}\left(x\right)W^{t}\phi_{G}\left(x\right)\right)$$

(4) $$y\left(x\right)=sign\left[\left(\underset{ijk}{\sum }\alpha_{i}\;y_{i\;}\phi_{G_{j}}\;\left(x_{i}\right)\phi_{G_{j}}\;\phi_{L_{k}}\;\left(x_{i}\right)\phi_{L_{k}}\;\left(x\right)\right)\right]$$

(5) $$y\left(x\right)=sign\left(W^{t}(\phi_{G}\left(x\right)\;⊗\;\phi_{L}\;\left(x\right))\right)$$

(6) $$y\left(x\right)=sign\left(\phi_{L}^{t}\left(x\right)W^{t}\phi_{G}\left(x\right)\right)$$

(7) $$y\left(x\right)=sign\left(W^{t}\phi_{G}\left(x\right)\right)$$

where $W_{k=}\sum_{i}\alpha_{i}\;y_{i\;}\phi_{L_{k}}\;\left(x_{i}\right)\phi_{G}\;\left(x_{i}\right)$, $\phi_{L_{k}}$ denote dimension k of $\phi_{L}\in R^{M}$, $W=\left[w_{1}\ldots \ldots w_{M}\right]$, $W\;\left(x\right)=W_{\phi_{L}}\left(x\right)$, and ⊗ is the Kronecker product. $\phi_{L}$ is the local feature space and $\phi_{G}$ is the global features space.

(8) $$\phi_{L_{k}}\;\left(x\right)=\tanh\left(\sigma \theta_{k}^{\prime t}\right)I_{k}\left(x\right)$$

while training the LD-SVM and smoothing the tree are shown in Figure 1, Equation (1) can further written as below:

(9) $$y\left(x\right)=\mathrm{sign}\left[\tanh\left(\sigma v_{1}^{t}x\right)w_{1}^{t}x+\;\mathrm{anh}\left(\sigma v_{2}^{t}x\right)w_{2}^{t}x)+\mathrm{anh}\left(\sigma v_{4}^{t}x\right)w_{4}^{t}x\right]\;$$

where $I_{k}\left(x\right)$ is the indicator function for each node k in the tree; θ is to go left or right; v stack with non-linearity; σ is sigmoid sharpness for the parameter scaling and could be set by validation. Higher values imply that the ‘tanh’ is saturated in the local kernel, while a lower value means a more linear range of operation for *θ*. The full optimization formula is given in Equation (10). The local deep kernel learning (LDKL) primal for jointly learning θ and W from the training data, where $\left\{{\left(x_{i},\;y_{i}\right)}_{i=1}^{N}\right\}$ can be described as:

(10) $$\underset{W,\theta ,\theta^{\prime }}{\min}P\left(W,\theta ,\theta^{\prime }\right)=\frac{\lambda_{w}}{2}T_{r}\left(W^{t}W\right)+\frac{\lambda_{\theta }}{2}T_{r}\left(\theta^{t}\theta \right)+\frac{\lambda_{\theta^{\prime }}}{2}T_{r}\left({\theta^{\prime }}^{t}\theta^{\prime }\right)+\overset{N}{\underset{i=1}{\sum }}L\left(y_{i},\phi_{L}^{t}\left(x_{i}\right)W^{t}x_{i}\right)$$

where $L=\max\left(0,1-\;y_{i},\phi_{L}^{t}\left(x_{i}\right)W^{t}x_{i}\right);\;\lambda_{w}$ is the weight of the regularization term; and $\lambda_{\theta }$ specifies the amount of space between the region boundary and the nearest data point to be left. $\lambda_{\theta^{\prime }}$ controls the curvature amount allowed in the model’s decision boundaries.

### 3.2. Decision Jungles

**Figure 2:**
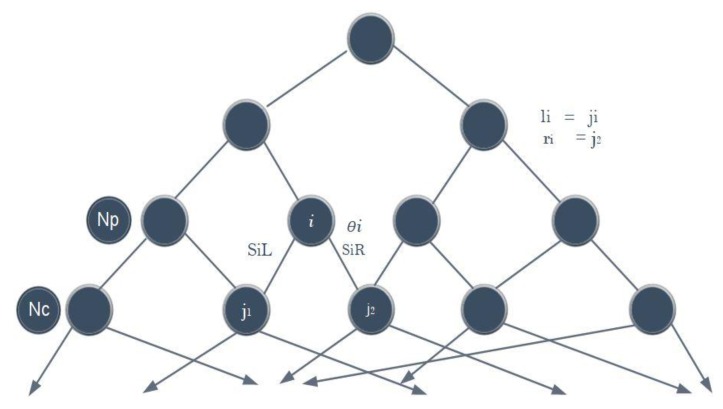
Decision jungles (DAGs).

Decision jungles are the latest addition to decision forests. They are comprised of a set of decision-making acyclic graphs (DAGs). Unlike standard decision trees, the DAG in the decision jungle enables different paths from root to leaf. A DAG decision has a reduced memory footprint and provides superior efficiency than a decision tree. Decision jungles are deemed as non-parametric models that provide integrated feature selection, classification, and are robust in the presence of noisy features. DAGs have the same structure as decision trees, except that the nodes have multiple parents. DAGs can limit the memory consumption by specifying a width at each layer in the DAG and potentially help to reduce overfitting [54]. Considering the nodes set at two consecutive levels of DAGs, Figure 2 shows that the nodes set consists of child nodes $N_{c}$ and parent nodes $N_{p}$. Let $\theta_{i}$ denote the parameters of the split function f for parent node $i\;\epsilon \;N_{p}$. $S_{i}$ denotes the categorized training samples (x,y), where it reaches node i, and set of samples can be calculated from node i, which travels through its left or right branches. Given $\theta_{i}$ and $S_{i}$, the left and right are computed by $S_{i}^{L}(\theta_{i})=\left(\left(x,y\right)\epsilon \;S_{i}|f\left(\theta_{i},x\right)\leq 0\right)$ and $S_{i}^{R}\left(\theta_{i}\right)=S_{i}/S_{i}^{L}\left(\theta_{i}\right)$, respectively. $l_{i}\;\epsilon \;N_{c}$ indicates the left outward edge from parental node $i\;\epsilon \;N_{p}$ to a child node, and $r_{i}\;\epsilon \;N_{c}$ denotes the right outward edge. Henceforth, the number of samples reaching any child node j ϵ N is given as:

(11) $$S_{j}\left(\left\{\theta_{i}\right\},\left\{l_{i}\right\},\left\{r_{i}\right\}\right)=\left[\underset{i\;\epsilon N_{p^{S\;.t\;.l_{i}=j}}}{\cup }S_{i}^{L}\left(\theta_{i}\right)\right]\cup \left[\underset{i\;\epsilon N_{p^{S\;.t\;.r_{i}=j}}}{\cup }S_{i}^{R}\left(\theta_{i}\right)\right]$$

### 3.3. CN2 Rule Induction

In this study, rule learning models were also explored for TSP. These models are usually used for classification and prediction solutions. The CN2 algorithm is a method of classification designed to induce simple efficiency; “if condition then predicts class,” even in areas where noise may occur. Inspired by Iterative Dichotomiser 3 (ID3), the original CN2 uses entropy as the function for rule evaluation; Laplace estimation may be defined as an alternative measure of the rule quality to fix unpleasant entropy (downward bias), and it is described as follows [67]:

(12) $$Laplace\;Estimation\;\left(R\right)=\;\frac{p+1}{P+n+k}$$

where ‘p’ represents the number of positive examples in the training set covered by Rule ‘R’; n represents the number of negative instances covered by R; and ‘k’ is the number of the training classes available in the training set.

### 3.4. Multi-Layer Perceptron

**Figure 3:**
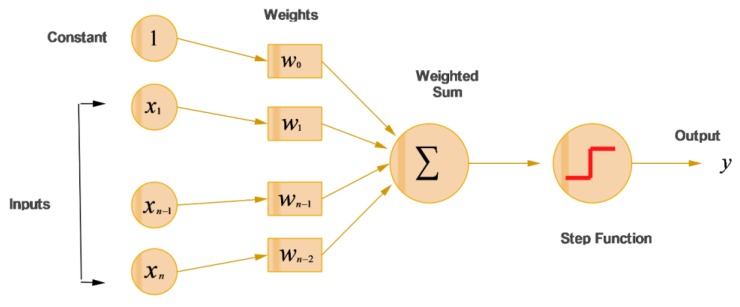
Schematic algorithm of multi-layer perceptron (MLP).

The most common ANN model is the multi-layer perceptron (MLP). In MLP, input values are transformed by activation function *f*, giving the value as an output from the neuron. The MLP is made up of various layers including one input layer, one or more hidden layers, and one output layer. In MLP, parameters such as the number of input variables, number of hidden layers, activation function, and learning rate play an important role in the design of neural network architecture. The multi-layer perceptron (MLP) is shown in Figure 3. Neurons have activation functions for both the hidden layer and the output layer; neurons receive only the input dataset and have no activation functions on the input layer. Weights are multiplied with inputs, and are summarized accordingly as;

(13) $$f\left(x_{i}\right)=\overset{n}{\underset{i=1}{\sum }}\left(w_{i}x_{i}\right)+bias$$

Whilst the most commonly applied activation function is logistic function (sigmoid function), given in following equation:

(14) $$f\left(x_{i}\right)=\frac{1}{1+e^{-x}}$$

## 4. Study Area

**Figure 4:**
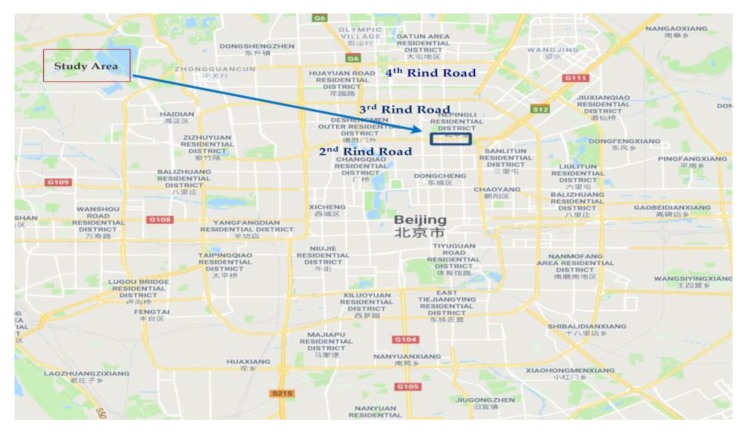
Second ring road (from Google Maps). Note: The Chinese words on map indicate names of surrounding infrastructure.

This study was conducted in the city of Beijing, China, which covers an area of 16,410 km^2^, and hosts 21.7 million people. Road transportation is an integral part of the city’s routine businesses, linking most households to workplaces or schools. There are 21,885 km of paved public road in Beijing (as of June 2016), 982 km of which are classified as highways [68]. According to the Beijing census, the number of private cars was close to 5.4 million, in addition to 5.3 million other vehicles in different categories including 330,100 trucks. The Second-Ring Road consists of six percent of the urban space of Beijing, with clusters of major companies, businesses, and administrative institutions, but generate 30% of the traffic volume per day [68,69]. Within this perspective, integrated urban planning is becoming difficult, so much so that 60% of the historical site of the city is lying on the Second Ring Road. Since the traffic hotspots are concentrated mainly in the center of Beijing, we have chosen an area as the study area at this location [68,70]. The Second Ring Road is approximately 33 km long including 37 on-ramps and 53 off-ramps. Figure 4 shows the study area on the Second Ring Road along with other different ring roads. In this study, a basic freeway segment of the Second Ring (L = 478.5 m) was selected.

### Data Collection and Parameters Setting

**Figure 5:**
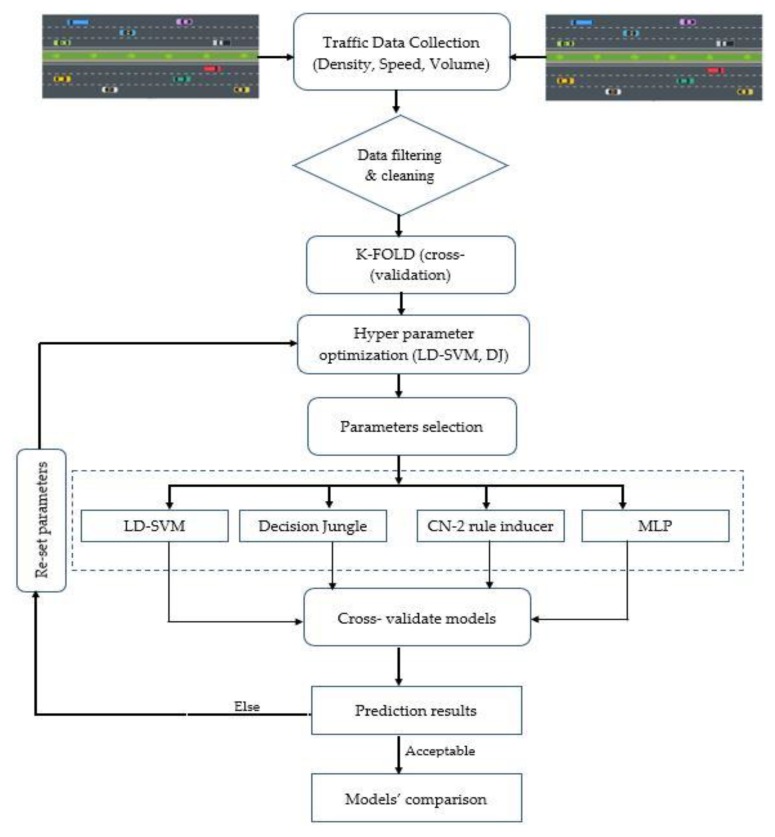
Proposed methodology for the study.

The first step in preparing the experiment was to develop a microscopic model using VISSIM (Micro Traffic Simulation Software) to capture all the essential data for the Second Ring Road. When simulating the field conditions, it is essential to calibrate the driving behavior parameters for the traffic simulator, and this was accomplished by standard procedures, as reported in the existing work [71]. In doing so, several simulation iterations were performed, incurring a different random seed to ensure that the model works under the real-time scenario. The proposed methodology for the present study is presented in Figure 5.

**Figure 6:**
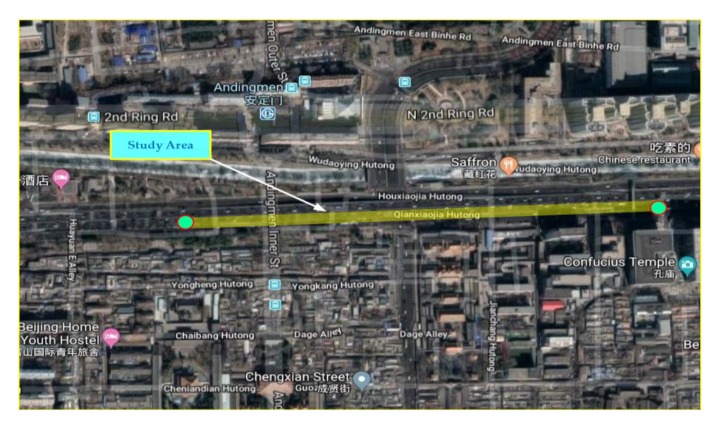
Basic freeway segment of the Second Ring Road (from Google Earth). Note: The Chinese words on map indicate names of surrounding infrastructure.

In this study, macroscopic traffic parameters (volume, speed, density) were obtained from the VISSIM simulation analysis. Traffic volume or flow rate can be defined as the number of vehicles that pass through a point on a highway or lane at a specific time, and is usually expressed in units of vehicles per hour per lane (v/h/l), while density is referred to the number of vehicles occupying a unit length of roadway, and is denoted by vehicles per km/mile per lane (v/m/ln). Occupancy is sometimes synonymously used with density; however, it should be noted that it shows the percentage of time that a road segment is occupied by vehicles. Traffic speed is another important state parameter, and can be found by the distance traversed per unit of time, and is typically expressed in km/h. or miles/h. These parameters are further calculated by using the link evaluation in VISSIM. Once the factual freeway architecture is achieved, the key macroscopic characteristics are identified in order to adjust the entire microscopic simulator (e.g., demand flow and split ratio). Demand flow is defined as the traffic volume as it utilizes the facility, while split ratio is the directional hourly volume (DHV) in the peak direction, which varies with respect to time, that is, the peak time and off-peak time. Additionally, the real traffic state of the Second Ring Road in this study was obtained from the Beijing Collaborative Innovation Center for Metropolitan Transportation. Thereby, the model of the road network deemed for the Second Ring Road was constructed by VISSIM. It has three lanes, where each lane is designated with an average width of 3.75 m, as shown in Figure 6. Simulations in the VISSIM were carried for 6 h, during the period 6:00 am to 12:00 pm, and a congested regime prevailed from 1.5 to 2 h (i.e., between 7:30 am to 9:30 am), leveraging the almost free flow for the remaining hours. Therefore, the transition state from D to F encountered few labels. Meanwhile, data were collected using different prediction horizons such as 5, 10, and 15 min.

**Figure 7:**
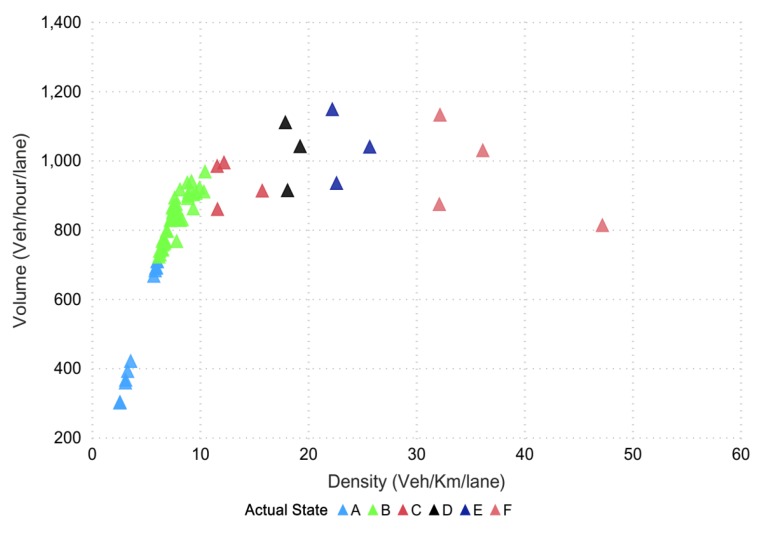
The actual density–flow via VISSIM.

To assess the freeway operations, level-of-service (LOS), a commonly used performance indicator, was used for qualitative evaluation purposes. The data collected from the VISSIM simulation was further divided into six levels [72], wherein the LOS defines the traffic state of each level. Traffic state is usually characterized by traffic-density on a given link, and is directly related with the number of vehicles occupying the roadway segment. It also represents the transient boundary conditions between two LOS levels. Moreover, to test the efficacy, classification models were built in python scripting orange software and azure machine learning to write the required procedures for extracting the traffic parameters, and level-of-service corresponded to highway capacity manual (HCM) [43,73]. The data points (in Figure 7) represent different points in time distributed spatially, which together define the LOS at the road segment. In the mentioned figure, different colors showed the states for 15 min, which is actually the LOS divided into six sub-levels based on density along the highway segment. We termed these levels as different states (from A to F) and further evaluated them for 5, 10, and 15 min intervals. Since stratified K-fold cross validation was opted to address the issue of imbalance data, the method aimed to choose the proportionate frequencies for each LOS class. Thus, it is likely that label D or any other label will be associated with true representative class. The actual density–flow captured on a segment of the Second Ring was simulated in VISSIM for a prediction horizon of 15 min and is shown in Figure 7.

## 5. Results and Discussion

### 5.1. K-Fold Cross-Validation

We selected the K-Fold cross-validation method (using k = 10), which is used for a better f-model, and it provides the appropriate settings for parameters. The original instances were randomly split into k equal parts. A single part was used for validation from the k split, and the remaining k minus one (k − 1) parts were used for the training set in order to develop the model. To do so, we revised the same technique k times. Each time a distinct validation dataset was selected, until the model’s final accuracy was equal to the average accuracy, that in turn, was achieved in each iteration. This technique has the advantage over repeated random sub-sampling as all the samples are used for training as well as in the validation, where each sample is used once for the validation. To avoid the problems of data imbalance and enhance the prediction accuracy of the proposed methods, several strategies have been suggested by previous studies. In this study, K-fold cross validation was used to overcome the issues and bias associated with imbalance and small datasets as the K-fold validation method is more efficient and robust compared to other conventional techniques, since it preserves the percentage of samples for each group or class. We tuned the parameters to obtain the best results with accuracy and they were selected using hyperparameter tuning.

### 5.2. Model Evaluation

In this study, we selected the most common evaluation metrics in order to assess the performances of the models known as F score and Accuracy. The F score is a measure of the accuracy of a test, also known as the F-1 score or F measure. The F−1 score is defined as the weighted average of recall and precision. To measure the overall performances of the model, the F-1 score was derived as follows:

(15) $$F-1=2\times \frac{Precison\;\times recall}{Precison+recall}$$

Accuracy is one of the classifications’ performance measures, which is defined as the ratio of the correct sample to the total number of samples as follows [74],

(16) $$Accuracy=\frac{TP+TN}{TP+TN+FP+FN}$$

where P and N denote the number of positive and negative samples, respectively. TP and TN indicate the true positive and true negative. FP and FN indicate the false positive and false negative, respectively.

### 5.3. Local Deep Kernel Learning SVM (LD-SVM)

**Figure 8:**
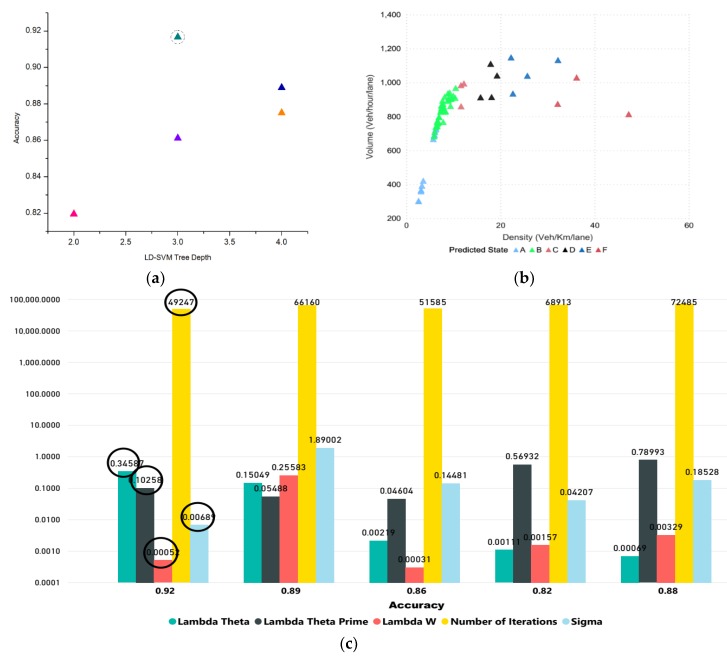
The LD-SVM model. (**a**) The impact of tree depth on accuracy. (**b**) Predicted state for next 15 min (**c**) Impact of Lambda theta, Lambda theta prime, Lambda W, and sigmoid function on accuracy.

The tuning parameters include LD-SVM tree depth, Lambda W, Lambda theta, Lambda theta prime, number of iterations, and sigmoid sharpness or sigma. Figure 8a shows the LD-SVM tree depth impact on accuracy and 92.00% accuracy was achieved when the tree depth was 3. The impact of the other parameters, Lambda W, Lambda theta, Lambda theta prime, number of iterations, and sigmoid sharpness or sigma, can be seen in Figure 8c. The best hyperparameter tuned values for these parameters were 0.00052, 0.34587, 0.1025, 49,247, and 0.0068, which were encircled and obtained using 10-fold cross-validation. Figure 8b shows the predicted state for the next 15 min

### 5.4. Decision Jungle

**Figure 9:**
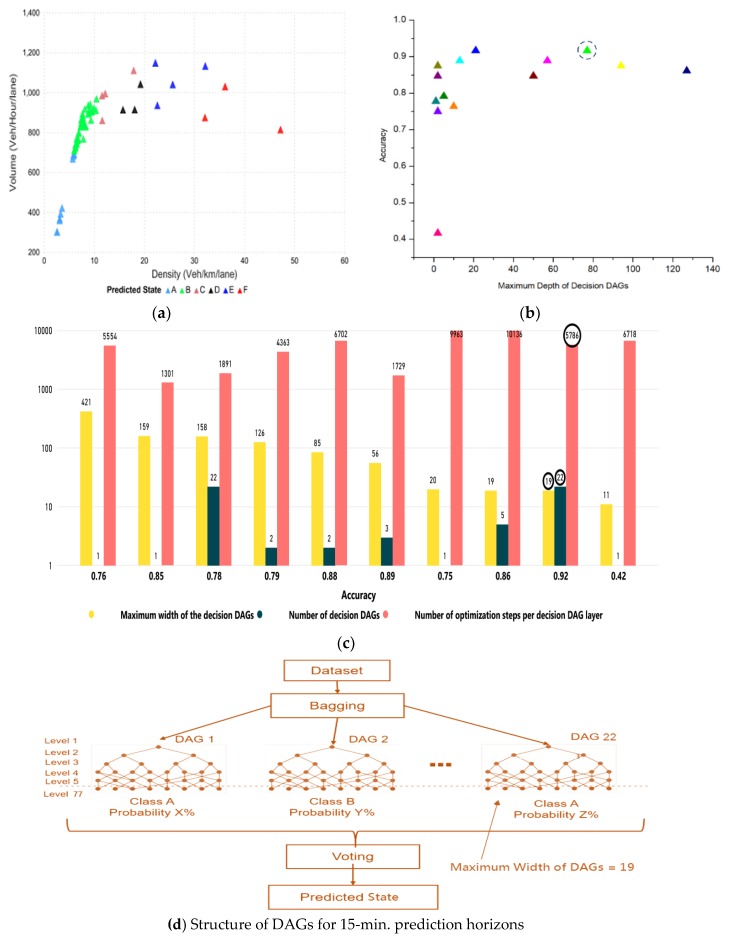
Decision jungle model. (**a**) The impact of maximum depth on accuracy. (**b**) Predicted state for the next 15 min (**c**) Impact of maximum width of the decision DAGs and number of decision DAGs on accuracy. (**d**) Number of DAGs, width, and depth of DAGs are shown for 15-min prediction horizons.

The tuning parameters in the decision jungle model were described by the maximum depth of the decision (DAGs), number of decision DAGs, number of optimization steps per decision, DAGs layer, and maximum width of the decision DAGs’. Figure 9b shows the impact of the maximum depth of decision DAGs on the accuracy of the model. The accuracy was 92% and was achieved when the maximum depth of the decision (DAGs) was 77. The best-tuned values for the other parameters are depicted in Figure 9c (such as the number of decisions DAGs, number of optimization steps per decision, and maximum width of decision DAGs’ were 22, 5786, and 19, respectively), and were obtained using 10-fold cross-validation. Since, our study considered 15 min prediction horizons as the structure of DAG is illustrated in Figure 9d, which shows the number of DAGs is 22 with a maximum depth of levels 77. The predicted state for 15 min horizons can be seen in Figure 9a.

### 5.5. CN2 Rule Induction

**Figure 10:**
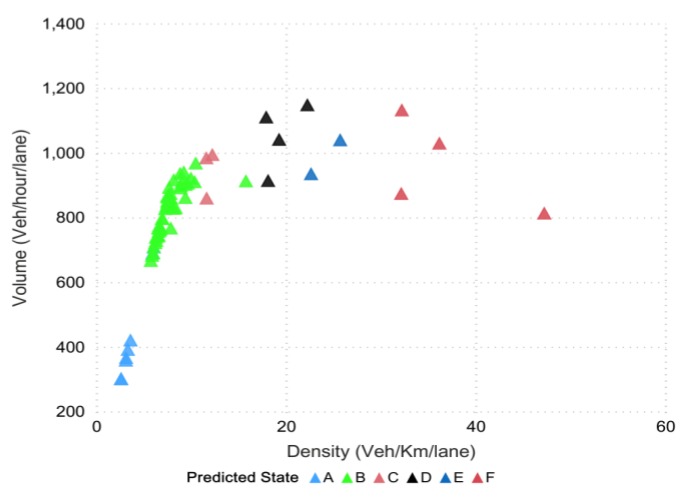
Predicted state for the next 15 min horizon.

CN2 utilizes a statistical significance test in order to ensure that the fresh rule represents a real correlation between features and classes. In fact, it is a pre-pruning technique that prevents particular rules after their implementation. Moreover, it performs a sequential covering approach at the upper stage (also defined as split-and-conquer or cover-and-remove), once used by the algorithm quasi-optimal (AQ) algorithm. The CN2 rule returns a class distribution in terms of the number of examples covered and distributed over classes. The distribution in Table 1 and the tables in the Appendix show that each number corresponded to the number of example(s) that belonged to class LOS = i, where i = {A, B, C, D, E, F} and “i” is the observed frequency distribution of examples between different classes. In another words, it represents number of the relevant class membership. The derived probabilities shown in Table 1 can be used to check the accuracy and efficiency of that particular rule. We adopted exclusive coverage in our implementation at the upper level such as unordered CN2 [62], whereas Laplace estimation was used for function evaluation at the lower level. Pre-pruning of rules was performed using two methods: (i) likelihood ratio statistic (LRS) tests, and (ii) minimum threshold for coverage of rules. The LRS test indicates two tests: first, a rule’s minimum level of significance α_1_, and the second LRS test is likened to its parent rule, as it checks whether the last rule specialization has a sufficient level of significance α_2_. The values for the LRS tests and rules for the different prediction horizons were obtained using 10-fold cross-validation. Figure 10 shows the predicted state for 15 min intervals. The values of α_1_ and α_2_ are listed in Table 2. The rule for the next 5 min and 10 min horizons is given in Appendix A, whilst the rule for the next 15 min horizons is given in Table 1.

### 5.6. Multi-Layer Perceptron (MLP)

**Figure 11:**
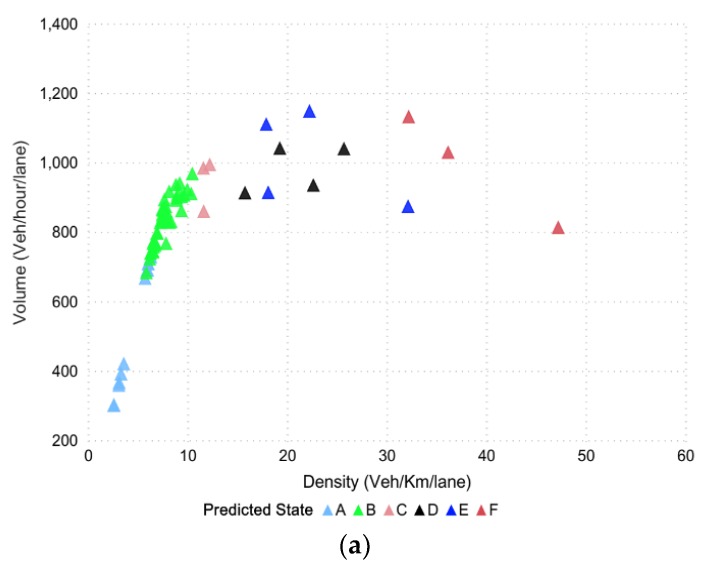

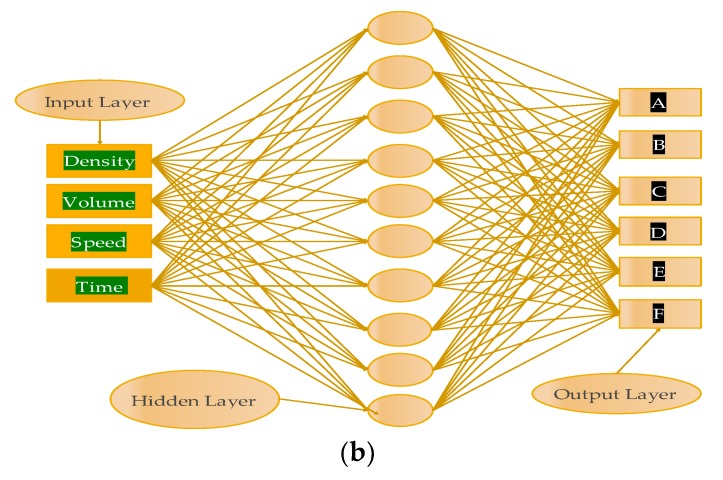
MLP model. (**a**) Predicted state for next 15 min horizon. (**b**) MLP network with 01 hidden layer.

In neural networks, learning includes adjusting the connection weights between neurons and each functional neuron’s threshold. We considered one input layer and one hidden layer with 35 neurons. The input layer had four nodes: speed, density, flow, and time duration (interval). The accuracy achieved using 10-fold cross-validation for different prediction horizons was compared (shown in Table 3) against the learning rate, momentum, activation function, and epochs. Figure 11a shows the predicted state for the next 15 min horizons. The input layer, hidden layers with neurons, and output layers for the MLP network are depicted in Figure 11b.

## 6. Model Comparison

**Figure 12:**
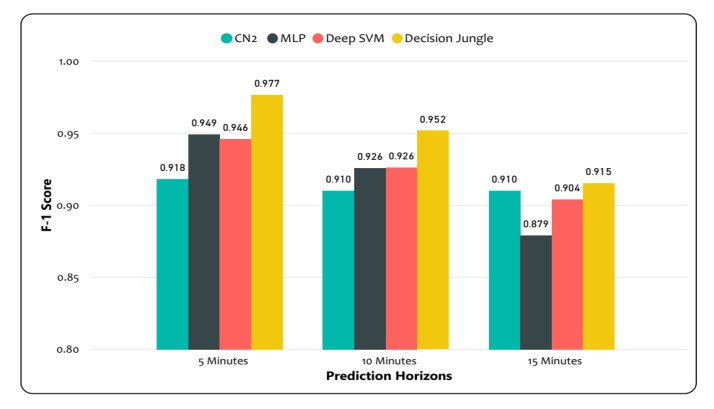
Model comparison. Weighted average F-1 score for decision jungles; weighted average F-1 score for LD-SVM; weighted average F-1 score for MLP; weighted average F-1 score for CN2 rule induction.

**Figure 13:**
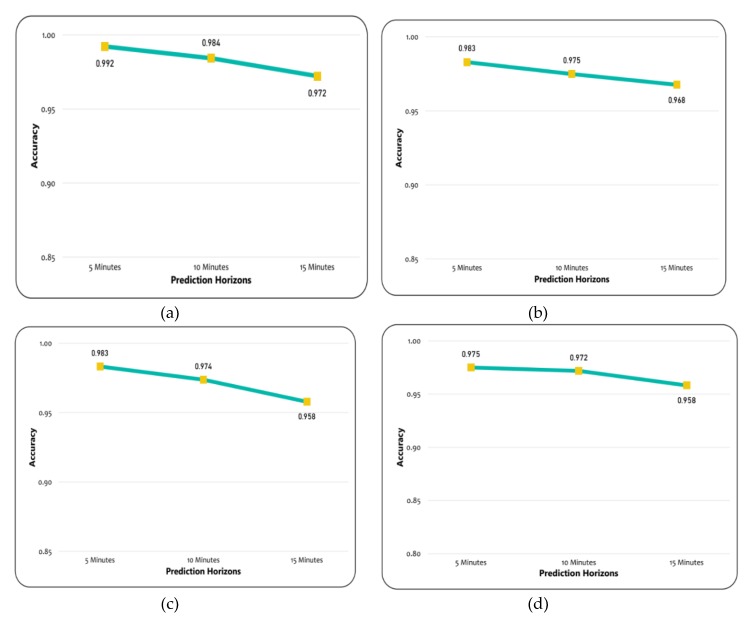
Model Comparison. (**a**) Accuracy for the decision jungles. (**b**) Accuracy for the LD-SVM. (**c**) Accuracy for the MLP. (**d**) Accuracy for the CN2 rule induction.

The weighted average F-1 score and accuracy were evaluated in order to assess the performances of different models. The results suggest that decision jungles outperformed the LD-SVM, CN2, and MLP, as shown in Figure 12. Additionally, the decision jungles and LD-SVM achieved a higher weighted average F-1 score. In particular, the decision jungle was found to have improved results over the LD-SVM, CN2, and MLP, and obtained high F-1 scores of 0.9777, 0.952, and 0.915 were predicated for time horizons of 15, 10, and 5 min, respectively. Similarly, the LD-SVM was slightly better than the MLP and CN2 as the F1-score was higher (0.904, 0.926, 0.946) for the 15, 10, and 5 min prediction horizons. However, the CN2 rule induction performed better, except for decision jungles, while the other models failed to achieve a higher F–1 score for the same prediction horizon. On the other hand, Figure 13a,b shows that decision jungles and LD-SVM also achieved higher accuracy when compared to the remaining models such as CN2 rule induction and MLP. It can be noted that as the prediction horizons increases, the F-1 score and accuracy decreases. This indicates that decision jungles were stable when compared to the results in accordance with time horizons of 15, 10, and 5. Unlike the LD-SVM, MLP and CN2 were found to be less effective at maintaining the stability of accuracy in different time horizons. However, the CN2 rule induction in Figure 13c,d) performed well and provided stable results only for the 10, and 15 min prediction horizons.

The experimental results are summarized in Table 4 and Table 5, where the models’ performances were computed using F-1 score and the average accuracy for different prediction horizons, respectively. It can be clearly seen that decision jungles achieved a higher F-1 score and gained a higher accuracy when compared to the other models for different prediction horizons. This shows that decision jungles achieved an average improvement of 95% and outperformed the remaining models. However, the LD-SVM performed better than the MLP and CN2 rule induction.

## 7. Conclusions

In this study, we improvised machine learning models with hyperparameter tuning optimization for short term TSP. Different schemes offered in parameter tuning were examined by performing the number of simulation iterations incurring different random seeds to ensure that the model worked efficiently under a real-time scenario. To do so, a comprehensive demonstration and the ability of different machine learning models were evaluated using different forecasting time-intervals at distinct time scales. The short-term traffic state was taken as a function of level-of-service (LOS) on a basic freeway segment along Second Ring Road in Beijing, China. Simulation of a transportation road demonstrated that decision jungles were more efficient and stable at different predicted horizons (time intervals) than the LD-SVM, MLP, and CN2 rule induction. Data utilized in this study was collected from traffic simulator VISSIM. Actual density–flow was captured on freeway segment via different prediction horizons of 15, 10, and 5 min. The experimental results showed and demonstrated the superior and robust performance of decision jungles compared to the LD-SVM, CN2 rule induction, and MLP. The overall performance of prediction results were improved by over 95 percent on average, which led to an accuracy of 0.982 and 0.975 for the decision jungle and LD-SVM. Moreover, the prediction performance for CN2 rule induction were also observed to be improved based on if–then rules in terms of the traffic patterns for different prediction horizons.

This study has some limitations that must be acknowledged. First, the proposed study was deployed in a developed urban freeway network model, so the simulated data need to be enhanced in future studies. Second, instead of justifying the efficacy of the suggested techniques using microscopic simulation platform via VISSIM, forthcoming studies may focus on investigating and verifying the performance of proposed methods with an improved model on real traffic data.

In the future, studies may focus on long-term traffic state prediction (hours, days, weeks), which could also be divided into different LOS groups. The study area can be extended from the basic freeway segment to weaving, merging, and diverging segments that cover the entire network range of the Second Ring Road. Studies could incorporate temperature, air quality, weather, and other external factors that are likely to affect travel demand, thus, enhance prediction accuracy. In addition, it could rely on considering larger and various types of traffic datasets to analyze various combinations of flow, occupancy, speed, and other characteristics of road traffic to improve the predictive accuracy by using improved machine learning methods for prediction and analytics.

## Figures and Tables

**Table 1:** Selected rules (for 15-min prediction horizon) with rule quality.

IF Condition	Then (Next State)	Distribution	Probabilities [%]	Rule Quality	Rule Length
Time (Seconds) ≤ 13500.0 AND Speed (Km/h) ≥ 117.83	A	[8, 0, 0, 0, 0, 0]	6: 4: 7: 7: 7: 7	0.903	2
Speed (Km/h) ≥ 88.43 AND Volume (Veh./h/lane) ≥ 723.35	B	[0, 45, 0, 0, 0, 0]	2: 90: 2: 2: 2: 2	0.98	2
Time (Seconds)≤9000.0 AND Density (Veh/Km/lane) ≥ 11.54	C	[0, 0, 3, 0, 0, 0]	11: 11: 44: 11: 11: 11	0.805	2
Density (Veh/Km/lane) ≤ 22.19 AND Density (Veh/Km/lane) ≥ 17.85	D	[0, 0, 0, 4, 1, 0]	9: 9: 9: 45: 18: 9	0.715	2
Speed (Km/h) ≥ 36.91 AND Density (Veh/Km/lane) ≥ 22.19	E	[0, 0, 0, 0, 3, 0]	11: 11: 11: 11: 44: 11	0.805	2
Density (Veh/Km/lane) ≥ 32.09	F	[0, 0, 0, 0, 0, 4]	10: 10: 10: 10: 10: 50	0.855	1

**Table 2:** CN2 rule setting parameter values.

Time Intervals (min)	$\alpha_{1}$	$\alpha_{2}$
**5**	0.05	0.03
**10**	0.05	0.02
**15**	0.05	0.03

**Table 3:** Configuration of the parameters for the multi-layer perceptron (MLP).

Prediction Horizons	Algorithm	Hidden Layers	Hidden Neurons	Activation Function	Epochs	Learning Rate	Momentum	Accuracy
5 min	MLP	01	35	Sigmoid	500	0.2	0.2	0.949
10 min	MLP	01	35	Sigmoid	500	0.2	0.2	0.924
15 min	MLP	01	35	Sigmoid	500	0.3	0.2	0.875

**Table 4:** F-1 score for the different model comparisons.

F-1 Score		Prediction Horizons (min)	
	5	10	15
Decision Jungle	0.976683061	0.951784174	0.915209941
LD-SVM	0.946083305	0.926083351	0.904265873
MLP	0.949	0.926	0.879
CN2 Rule Induction	0.92	0.91	0.910

**Table 5:** Accuracy for different models comparisons.

Accuracy		Prediction Horizons (min)	
	5	10	15
Decision Jungle	0.992212	0.984277	0.972222
LD-SVM	0.982866	0.974843	0.967593
MLP	0.983262872	0.973	0.9577
CN2 Rule Induction	0.975	0.97183	0.9581

## Appendix A

**Table A1:** If–then rules for the next 5-min horizon.

IF Conditions	THEN (Next State)	Distribution	Probabilities [%]	Rule Quality	Rule Length
Density (Veh/Km/lane) ≤ 7.03	A	[95, 1, 0, 0, 0, 0]	94: 2: 1: 1: 1: 1	0.98	1
Speed (Km/hr) ≥ 105.65 AND Volume (veh/h/lane) ≥ 847.03	B	[0, 38, 0, 0, 0, 0]	2: 89: 2: 2: 2: 2	0.975	2
Density (Veh/Km/lane) ≥ 7.03 AND Speed (Km/h) ≥ 85.03	B	[0, 39, 1, 0, 0, 0]	2: 87: 4: 2: 2: 2	0.952	2
Density (Veh/Km/lane) ≥ 11.45 AND Speed (Km/h) ≥ 64.05	C	[0, 0, 9, 0, 0, 0]	7: 7: 67: 7: 7: 7	0.909	2
Density (Veh/Km/lane)≤ 22.26 AND Density (Veh/Km/lane) ≥ 16.84	D	[0, 0, 0, 7, 1, 0]	7: 7: 7: 57: 14: 7	0.8	2
Speed (Km/h) ≥ 37.3 AND Density (Veh/Km/lane) ≥ 22.26	E	[0, 0, 0, 0, 3, 0]	11: 11: 11: 11: 44: 11	0.8	2
Density (Veh/Km/lane) ≥ 29.64	F	[0, 0, 0, 0, 0, 19]	4: 4: 4: 4: 4: 80	0.952	1

**Table A2:** If–then rules for the next 10-min horizon.

IF Conditions	THEN (Next State)	Distribution	Probabilities [%]	Rule Quality	Rule Length
Time (Seconds) ≤ 8400.0 AND Speed (Km/h) ≥ 118.5	A	[14, 0, 0, 0, 0, 0]	75: 5: 5: 5: 5: 5	0.938	2
Density (Veh/Km/lane) ≥ 6.04	A	[7, 1, 0, 0, 0, 0]	57: 14: 7: 7: 7: 7	0.8	1
Speed (Km/h) ≥ 87.12 AND Density (Veh/Km/lane) ≥ 6.04	B	[0, 56, 0, 0, 0, 0]	2: 92: 2: 2: 2: 2	0.983	2
Density (Veh/Km/lane) ≤ 16.59 AND Density (Veh/Km/lane) ≥ 11.01	C	[0, 0, 12, 1, 0, 0]	5: 5: 68: 11: 5: 5	0.867	2
Density (Veh/Km/lane) ≤ 23.65 AND Density (Veh/Km/lane) ≥ 16.59	D	[0, 0, 0, 6, 1, 0]	8: 8: 8: 54: 15: 8	0.778	2
Speed (Km/h) ≥ 43.9 AND Density (Veh/Km/lane) ≥ 23.65	E	[0, 0, 0, 0, 2, 0]	12: 12: 12: 12: 38: 12	0.75	2
Density (Veh/Km/lane) ≥ 28.42	F	[0, 0, 0, 0, 0, 7]	8: 8 :8 :8 :8 :62	0.889	1
